# A System of Anatomy, for the Use of Students of Medicine

**Published:** 1843-02-04

**Authors:** 


### BIBLIOGRAPHICAL NOTICE.

*A system of Anatomy, for the use of Students of Medicine.* By Caspar Wistar, M. D., late Professor of Anatomy in the University of Pennsylvania. *With Notes and Additions.* By W. E. Horner, M. D., Professor of Anatomy in the University of Pennsylvania. Eighth Edition. *Entirely remodelled, and Illustrated with more than two hundred engravings.* By J. Pancoast, M. D., Professor of General, Descriptive, and Surgical Anatomy in the Jefferson Medical College, etc., etc. In two volumes. Philadelphia: 1842. 8vo. pp. 538, 622.

The favourable reception of the last edition of Professor Wistar's Anatomy, enlarged and illustrated, has induced the publication of the present one. The additions of Professor Horner are retained, and the work has been entirely remodelled by Professor Pancoast. The amount of original matter added is so great and of such importance, as to give it almost the character of an original work. We confess that we are no admirers of these mosaic productions, and we are sorry that Dr. Pancoast did not favour us with a treatise of his own. We regret this the more from the ability which he has displayed in the discharge of his editorial duties. He has placed the present work quite *au niveau* of the actual state of anatomical science. His chief additions have been made to the departments of general and microscopic anatomy, and he has here displayed much research and judgment. The present edition is illustrated with eight coloured copper-plate engravings of the blood-vessels, and upwards of two hundred and twenty wood-cuts, and its general appearance is highly creditable.

